# Assessment of Alterations in Permanent Premolars After Endodontic Treatment of Predecessor Primary Molars: A Prospective Study

**DOI:** 10.1111/ipd.70024

**Published:** 2025-08-01

**Authors:** Rafaela Lourdes de Sousa, Elisa Varela de Oliveira, Josiane Pezzini Soares, Nashalie Alencar, Jéssica Barasuol, Pablo Silveira Santos, Mariane Cardoso, Michele Bolan

**Affiliations:** ^1^ Department of Dentistry Federal University of Santa Catarina (UFSC) Florianópolis Brazil

**Keywords:** deciduous tooth, developmental defects of enamel, ectopic tooth eruption, endodontics

## Abstract

**Background:**

The relationship between primary teeth and their successors involves complex interactions that may influence the development of permanent teeth.

**Aim:**

To evaluate alterations in permanent premolars after endodontic treatment of their primary molar predecessors.

**Design:**

A prospective study was conducted with children initially aged 5 to 9 years, who were submitted to pulpectomy of a primary molar. The protocol involved instrumentation of root canals and obturation with a zinc oxide‐eugenol paste. Follow‐up examinations were performed until exfoliation of the primary molars and full eruption of the successor premolar. Permanent premolars were assessed for developmental defects of enamel, ectopic eruption, and crown rotation. Logistic regression was used to explore variables associated with these outcomes.

**Results:**

Forty‐seven children were followed up for approximately 6 years. Among the successor permanent premolars, 21.3% presented crown rotation, 10.6% ectopic eruption, 6.4% enamel hypomineralization, and 2.1% enamel hypoplasia. No statistically significant association was observed between the assessed alterations and demographic or treatment‐related variables, including pulp status, interradicular lesion, root resorption, or root overfilling (*p* > 0.05).

**Conclusion:**

Eruptive alterations were the most frequent findings. No association was identified between variables related to the endodontic treatment of primary molars and subsequent clinical alterations in permanent premolars.

**Summary**

- Why this paper is important to pediatric dentists
  ○ Provides long‐term clinical evidence on developmental and eruption alterations of permanent successor teeth after endodontic treatment of their predecessor.
  ○ Clarifies that common treatment‐ and tooth‐related factors may not be associated with developmental or eruptive alterations.
  ○ Emphasizes the importance of preserving primary teeth through appropriate pulp therapies to maintain dental arch integrity and avoid premature tooth loss complications.

## 1 Introduction

The occurrence of early childhood caries and traumatic dental injuries is the main cause leading to pulpal damage, which affects a significant number of children in the primary dentition phase [1]. These conditions often lead to repercussions for the permanent successor tooth germ due to its close proximity to the affected primary tooth [2]. Such consequences typically arise from pulpal infection [2] or the direct impact of trauma [3, 4]. When apical periodontitis in primary teeth extends to the developing permanent germ, it may interfere with odontogenesis, resulting in a range of sequelae, including enamel hypomineralization or hypoplasia, arrested development of the permanent tooth, formation of dentigerous cysts, germ expulsion resembling bone sequestration in osteomyelitis, and alterations in the follicular structure [1, 5, 6]. These changes may affect the reduced enamel epithelium, the gubernacular canal, and ultimately disrupt the eruption process [7].

Pulp therapy in primary teeth aims to preserve the tooth in the oral cavity until its natural exfoliation, thereby maintaining space for the permanent successor and supporting the proper development of the dental arch [8, 9]. Moreover, early loss of primary teeth can negatively impact esthetics and function in young children, ultimately affecting their quality of life [10]. Pulpectomy is indicated when clinical and radiographic signs suggest irreversible pulpitis or pulp necrosis [11]. Therefore, selecting and properly executing the appropriate technique must be guided by an accurate diagnosis and careful consideration of the pulp's current condition [12].

The success of endodontic treatment in primary teeth relies on the effective reduction or elimination of bacteria, not only from the root canals but also from areas inaccessible to chemical–mechanical preparation [11, 12]. The complex morphology of primary teeth, particularly the pronounced root canal curvatures, poses significant challenges to instrumentation [13]. Additionally, the presence of irregular root resorption further complicates the determination of a precise apical limit for both instrumentation and obturation [13].

In addition to the use of an appropriate technique, the success of pulpectomy in primary teeth depends on the choice of an ideal obturation material. Such material should meet several criteria, including biocompatibility with periapical tissues and the developing permanent tooth germ, absorbability when extruded beyond the apex, a resorption rate similar to that of the primary tooth root, antiseptic properties, good adhesion to canal walls, ease of application and removal, radiopacity, and the absence of tooth discoloration [14, 15]. However, no existing material currently satisfies all these requirements [14]. The most used root canal filling materials in pediatric dentistry include zinc oxide‐eugenol (ZOE) paste, iodoform‐based pastes, and calcium hydroxide‐based formulations [11, 14, 16]. While some reports suggest that ZOE may have a higher clinical success rate and is widely used among pediatric dentists, other studies have not demonstrated clear superiority over alternative materials [11, 14, 17]. Overfilling with ZOE has been associated with potential alterations in the eruption path of the permanent successor tooth and exhibits slower resorption than primary roots [18, 19].

Considering the scarcity of long‐term evidence evaluating alterations in permanent teeth following the exfoliation of endodontically treated primary molars, longitudinal and prospective studies are essential to further investigate potential repercussions on the development, eruption patterns, and structural integrity of successor teeth. Therefore, the primary objective of this study was to assess developmental and eruptive alterations in permanent premolars after endodontic treatment of their primary molar predecessors. Additionally, potential demographic, tooth‐related, and treatment‐related variables were investigated as possible explanatory factors associated with these alterations in the successor premolars.

## 2 Materials and Methods

The present manuscript was reported following the recommendations adapted from the CONSORT (CONsolidated Standards Of Reporting Trials) Statement [20].

### 2.1 Trial Design, Setting, and Ethical Considerations

The present prospective study involved a longitudinal assessment of patients enrolled in three randomized controlled trials conducted within the Postgraduate Program in Dentistry at Universidade Federal de Santa Catarina (Federal University of Santa Catarina, Brazil). The studies' protocols were submitted and approved by the local Ethics Committee for Research involving Human Subjects under protocol numbers 1.450.709, 2.308.475, and 2.595.678. In addition, the protocols were registered in the Clinical Trials database (code NCT03161639) and in the Brazilian Registry of Clinical Trials (ReBEC) under registration codes RBR‐7FPGZG and RBR‐3T597F. Participants were only enrolled in the study after the children assented to their participation and their legal guardians have signed an informed consent form.

### 2.2 Eligibility Criteria

All 197 patients enrolled in the previous studies were considered eligible for inclusion. Each patient had undergone endodontic treatment on one primary molar. However, individuals who received orthodontic treatment or any restorative procedure involving the successor premolar were excluded from the analysis.

### 2.3 Endodontic Treatment Protocol

Endodontic treatments were performed in a single session by three trained PhD candidates in Pediatric Dentistry (N.A., J.B., and J.P.S.). Prior to treatment, participants underwent clinical and radiographic assessment to evaluate the indicated primary molar and adjacent structures, to identify signs such as fistula, abscess, edema, or interradicular lesion and pathological resorption. Eligible children, ages ranging from 5 to 9 years, returned for treatment between 2016 and 2018. The procedures were performed following local anesthesia with lidocaine 2% with epinephrine 1:100000 (Alphacaine, Nova DFL, Rio de Janeiro, Brazil), after rubber dam placement. Root canals were instrumented using manual *k‐*files (Dentsply Maillefer, Ballaigues, Switzerland) or rotary files (ProDesign Logic, Easy *Equipamentos Odontológicos*, Belo Horizonte, Brazil), irrigated with 1% sodium hypochlorite (Rioquímica, São Paulo, Brazil), and filled with ZOE paste (Biodinâmica, Rio de Janeiro, Brazil).

### 2.4 Outcome and Independent Variables

The primary outcome of this investigation was the occurrence of developmental or eruption‐related alterations in permanent premolars. Developmental enamel defects were diagnosed following the criteria established by Ghanim et al. (2015) [21]. Hypomineralization was identified as a demarcated opacity greater than 1 mm in diameter, presenting as white, creamy, or brown discoloration. Hypoplasia was defined as the presence of pits, grooves, or areas of partial or complete enamel loss, characterized by rounded, smooth borders adjacent to the surrounding intact enamel.

Additionally, ectopic eruption was defined as the mispositioning of permanent premolars that failed to achieve proper occlusion, while crown rotation referred to the rotation of the tooth around its longitudinal axis [22]. Radiographs taken during the follow‐up periods were also evaluated to assist the detection of deviation of the eruption path of the permanent successor. Radiographic imaging was standardized using a Rinn pediatric radiography positioning device (Dentsply, Elgin, IL, USA) and size 0 Insight radiographic film (Carestream/Kodak). The X‐ray beam was positioned perpendicular to the film. To ensure reproducibility and minimize distortion in follow‐up radiographs, a condensation silicone bite registration was affixed to the Rinn device. Radiographs were acquired using X‐ray machines set at 70 kVp and 10 mA, with exposure time adjusted according to the specific tooth. Film processing was performed in accordance with ambient temperature conditions.

The main examiner, a master's student in Pediatric Dentistry (RLS), was trained by a Pediatric Dentistry professor (MB) with over 20 years of clinical experience. The training included theoretical instruction and calibration exercises using intraoral photographs illustrating different developmental enamel defects, eruption disturbances, and unaffected conditions. To minimize potential bias, the clinical assessment of alterations was conducted by consensus between the two examiners.

Independent variables included demographic characteristics (patient sex and age at the time of endodontic treatment) as well as treatment‐ and tooth‐related factors. Dental records and periapical radiographs of the treated primary teeth were reviewed to collect data on the following variables: pulp status (irreversible pulpitis or pulp necrosis), presence of interradicular lesions, pathological root resorption, rupture of the follicular bone crypt of the successor permanent premolar, and root filling extravasation. These data were independently extracted and cross‐verified by both examiners (R.L.d.S. and M.B.) to ensure precision and consistency.

### 2.5 Statistical Analysis

Data analysis was conducted using the Statistical Package for Social Sciences (SPSS for Windows, version 24.0, SPSS Inc.). Initially, a descriptive analysis was performed to characterize the sample. Contingency tables and a logistic regression model were then used to explore potential associations between demographic or treatment‐related variables and the outcome. Crude odds ratios (OR) and 95% confidence intervals (CI) were calculated. Two independent variables, dental pulp status and rupture of the follicular bone crypt, were excluded from the logistic regression model due to zero cell counts in their bivariate association with the outcome, indicating complete or quasi‐complete separation, which led to infinite coefficient estimates. Since no independent variable demonstrated a statistically significant association with the outcome (*p* > 0.20), an adjusted multivariable model was not constructed.

Additionally, *post hoc* power analysis was performed using G*Power software (version 3.1.9.2), considering an effect size of 0.15 and a significance level (*α*) of 0.05. The achieved power of the sample was 63%.

## 3 Results

**FIGURE 1:**
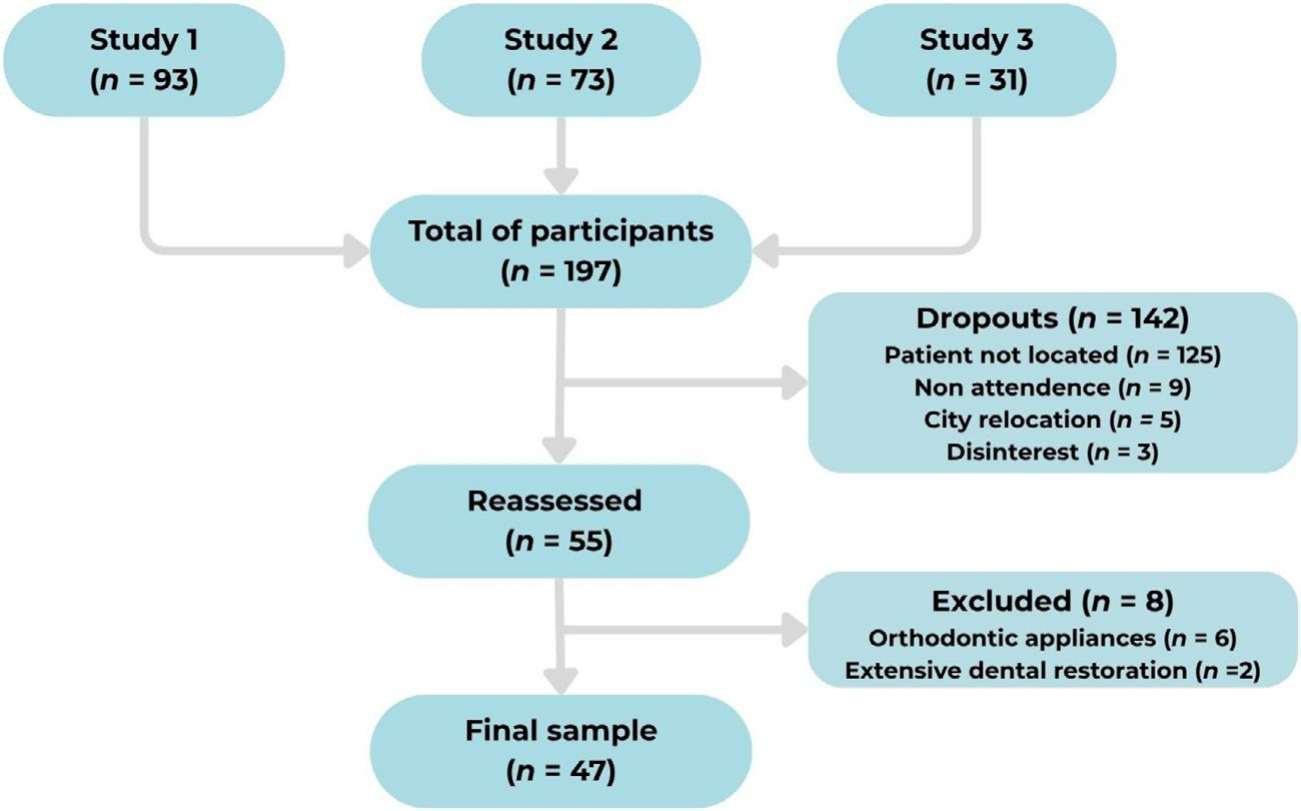
Flowchart presenting an overview of patient enrollment and follow‐up processes.

The final sample of the present study consisted of 47 patients. Figure 1 illustrates the patient enrollment process. Of the 197 initially eligible patients, 142 were classified as dropouts, with the most common reason being failure to locate the patient (*n* = 125). Following the reassessment of 55 patients, eight were excluded from the analysis due to the use of orthodontic appliances (*n* = 6) or the presence of extensive restorations in the permanent successor tooth (*n* = 2).

Table 1 presents the main characteristics of the included sample. The majority of participants were boys (*n* = 27), with ages ranging from 5 to 9 years at the time of endodontic treatment and 11 to 15 years when the successor premolar was evaluated, resulting in a mean follow‐up of 6 years. Most treated teeth exhibited pulp necrosis (93.6%) and interradicular lesions (85.1%), whereas pathological root resorption was observed only in 14.9% of cases.

**TABLE 1:** Main characteristics of the sample (*n* = 47).

Demographic characteristics	*n*	%
Sex		
Girls	20	42.6
Boys	27	57.4
Age (mean ± SD)	6.87 ± 1.05	
Tooth characteristics		
Dental pulp state		
Irreversible pulpitis	3	6.4
Pulp necrosis	44	93.6
Interradicular lesion		
Absent	7	14.9
Present	40	85.1
Pathological root resorption		
Absent	40	85.1
Present	7	14.9
Rupture of follicular bone crypt		
Absent	45	95.7
Present	2	4.3
Overfilling		
Absent	30	63.8
Present	17	36.2

Abbreviation: SD: standard deviation.

**FIGURE 2:**
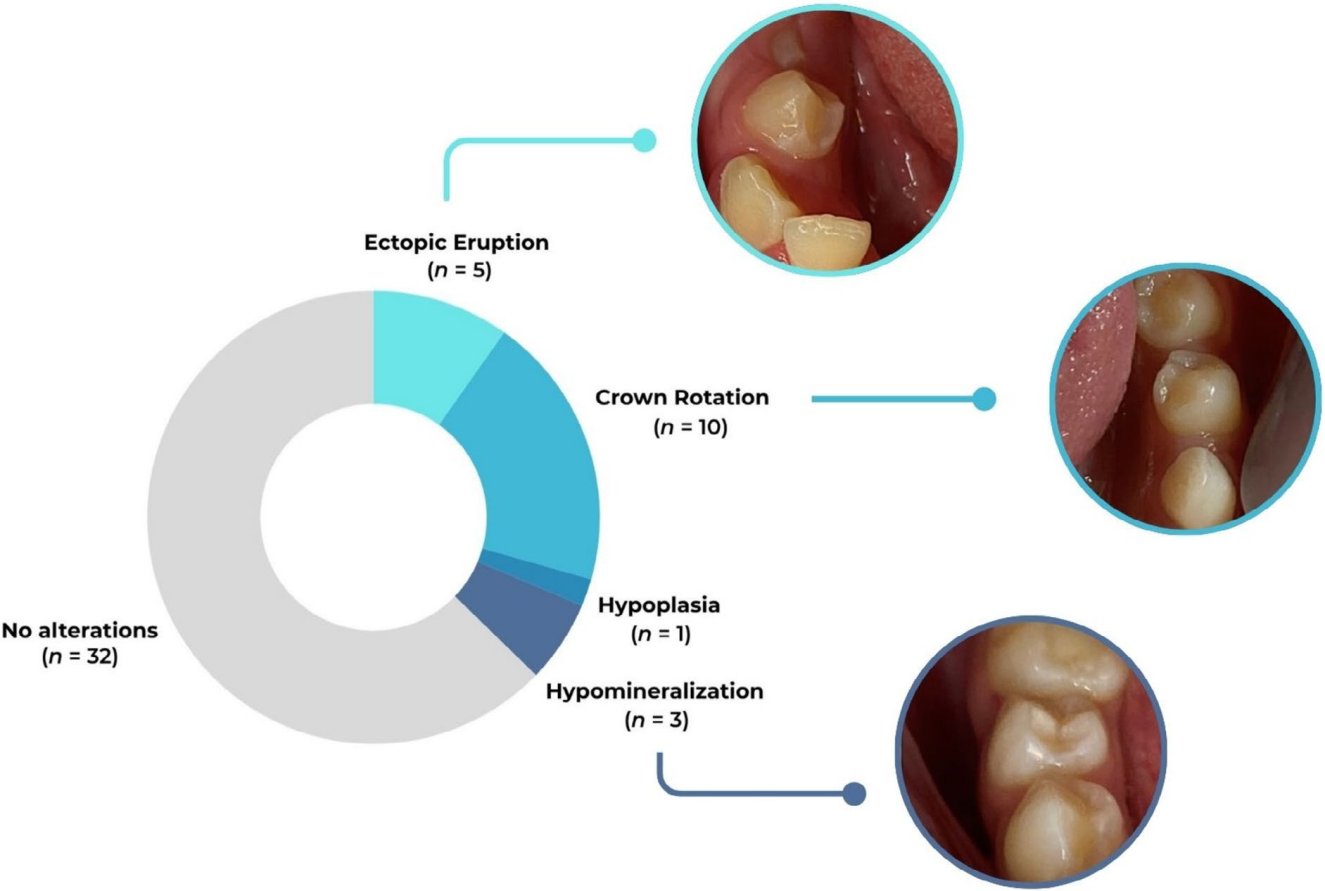
Frequency and clinical examples of developmental and eruptive alterations observed in permanent premolars following endodontic treatment of the predecessor primary molars.

Figure 2 displays the frequency of developmental and eruption‐related alterations in the permanent successor premolars. Crown rotation was the most frequently observed alteration (*n* = 10), followed by ectopic eruption (*n* = 5), enamel hypomineralization (*n* = 3), and hypoplasia (*n* = 1). Thirty‐two teeth presented no alterations, while four exhibited two alterations simultaneously (crown rotation and ectopic eruption).

Furthermore, Table 2 presents the contingency tables and results of the crude logistic regression analysis. None of the demographic or treatment‐related variables, including pulp status, interradicular lesion, root resorption, or root filling extravasation, demonstrated a statistically significant association with the occurrence of alterations in the successor premolars (*p* > 0.05).

**TABLE 2:** Contingency tables and logistic regression model to assess possible variables associated with the occurrence of alterations in permanent premolars (*n* = 47).

Independent variables	Premolars' alterations *n* (%)		Total	*p*	OR_c_	*p*
	Absent	Present				
Sex						
Girls	15 (75.0)	5 (25.0)	20	0.38^a^	1	0.38
Boys	17 (53.0)	10 (37.0)	27		0.57 (0.16–2.03)	
Age						
5 to 7 years	24 (68.6)	11 (31.4)	35	0.58^b^	1	0.90
8 and 9 years	8 (66.7)	4 (33.3)	12		0.91 (0.23–3.70)	
Dental pulp state						
Irreversible pulpitis	3 (100)	0 (0)	3	0.54^b^	—	—
Pulp necrosis	29 (65.9)	15 (34.1)	44			
Interradicular lesion						
Absent	5 (71.4)	2 (26.6)	7	0.61^b^	1	0.84
Present	27 (67.5)	13 (32.5)	40		0.83 (0.14–4.87)	
Pathological root resorption						
Absent	27 (67.5)	13 (32.5)	40	0.61^b^	1	0.84
Present	5 (71.4)	2 (28.6)	7		1.20 (0.20–7.05)	
Rupture of follicular bone crypt						
Absent	30 (66.7)	15 (33.3)	45	0.46^b^	—	—
Present	2 (100)	0 (0)	2			
Overfilling						
Absent	21 (70.0)	9 (30.0)	30	0.71^a^	1	0.71
Present	11 (64.7)	6 (35.3)	17		0.77 (0.22–2.78)	

Abbreviation: ORc: crude (unadjusted) odds ratio.

^a^ Pearson's chi‐squared test.

^b^ Fisher's exact test.

## 4 Discussion

The main findings of the present study indicate that eruptive disturbances, such as crown rotation and ectopic eruption, were the most frequently observed alterations in permanent premolars following endodontic treatment of the corresponding primary molars. Furthermore, no statistically significant associations were found between the observed alterations and the demographic, treatment‐related, or tooth‐related variables analyzed.

The intimate anatomical relationship between primary teeth and the follicular tissues of their permanent successors involves complex biological interactions that may influence the development of the succeeding teeth. Chronic infection in a primary tooth can extend to the follicle of the developing permanent tooth, potentially causing inflammation [2, 6, 7]. Previous studies [2, 5, 6, 7] have demonstrated that chronic apical periodontitis in primary teeth may adversely affect the growth, development, and eruption of permanent teeth, resulting in a spectrum of disturbances such as enamel hypoplasia, hypomineralization, abnormal tooth morphology, ectopic eruption, and, in severe cases, odontogenic cyst formation that may lead to necrosis of the permanent tooth germ.

In this sense, alterations in the permanent successor tooth appear to be primarily associated with the pre‐existing infection rather than the endodontic procedure itself [2, 6, 23]. By addressing the infection in the periradicular tissues, endodontic treatment of primary teeth plays a crucial role in preserving the integrity of the developing stomatognathic system. Maintaining the primary tooth not only pauses disease progression but also helps prevent a range of complications, including masticatory dysfunction, loss of arch space, eruptive alterations, speech impairments, and psychological issues related to early loss of primary teeth.

Particular concern has been raised regarding the use of ZOE paste as a root canal filling material in primary teeth, especially in cases of overfilling [18, 19]. It has been reported that ZOE exhibits a slower resorption rate compared to that of the primary tooth roots [18, 19]. This discrepancy may interfere with the natural exfoliation process and may have the potential to alter the eruption pathway of the permanent successor tooth. However, these assertions are based on evidence of limited robustness and were not observed in the present study.

Additionally, a previous study [23] investigating the association between radiographic rupture of the follicular bone crypt and enamel alterations in premolars reported no significant correlation between these variables, corroborating our findings. Interestingly, age over 6 years at the time of intervention was associated with a higher occurrence of enamel alterations, suggesting that delayed treatment may increase the exposure of the developing permanent tooth to inflammatory byproducts from interradicular lesions [23]. However, the stage of calcification of the developing tooth germ is also a critical factor to consider [23]. Younger patients, whose permanent teeth are in earlier stages of development, may be more susceptible to structural alterations due to the greater vulnerability of immature dental tissues [23, 24].

A major strength of this study is its prospective design with long‐term follow‐up, allowing for direct observation of outcomes from the time of endodontic treatment in primary molars until the exfoliation of these teeth and the subsequent eruption of their permanent successors. This approach provides a robust temporal link between the intervention and the outcome, which is seldom achieved in studies on this topic. Unlike many investigations that are retrospective in nature or rely solely on radiographic data, this study combined standardized clinical and radiographic assessments. Alterations in the permanent teeth were diagnosed using examiner consensus, enhancing diagnostic reliability and minimizing detection bias.

However, some relevant limitations must be acknowledged. The final sample size was relatively small, primarily due to a high attrition rate over the extended follow‐up period, which limited the statistical power of the analysis and may have hindered the detection of associations. Although high dropout rates are expected in long‐term studies, this issue was exacerbated by the COVID‐19 pandemic. During this period, many caregivers changed their contact information or relocated to different cities, making it difficult to re‐establish contact, contributing to the reduced sample. Additionally, the absence of a control group, such as the contralateral tooth, restricted comparative analysis. Although initially considered, the use of the contralateral tooth was ultimately not feasible, as many had not yet fully erupted. Furthermore, despite the standardization of treatment and radiographic procedures, variations in clinical execution by different postgraduate students and the inherent biological variability among patients may have introduced some heterogeneity. Finally, although the examiners underwent specific training to identify developmental and eruptive alterations, no formal calibration process was conducted. To mitigate potential detection bias, all clinical examinations were performed by consensus between the two examiners.

In summary, caution is warranted when interpreting the findings of this study. The results have limited external generalizability, as the sample was drawn from a single geographic location, and the high dropout rate may have reduced the power to detect significant associations. Despite these limitations, the study offers valuable insights into the long‐term outcomes of pulpectomy in primary molars and its potential impact on the development and eruption of successor permanent premolars, particularly due to its prospective longitudinal design.

Future studies should aim to include larger and more diverse samples to improve statistical power and the generalizability of findings. Efforts to reduce attrition over long follow‐up periods, such as maintaining updated contact information or utilizing digital follow‐up platforms, may help retain participants. Incorporating a control group, ideally using the contralateral tooth when feasible, would strengthen comparative analyses. Additionally, longitudinal studies with multi‐center collaboration could help validate the present findings across different populations and clinical settings.

In conclusion, nearly one‐third of the permanent premolars exhibited alterations following the exfoliation of endodontically treated predecessor primary molars. Eruptive alterations, such as crown rotations and ectopic eruptions, were the most frequently observed, whereas developmental enamel defects were less common. No significant associations were identified between the occurrence of these alterations and the evaluated tooth‐ or treatment‐related variables.

## Author Contributions

R.L.d.S. and E.V.O.: contributed to the acquisition of data, analysis and interpretation of data, drafting the article, revising it critically, and final approval of the version to be published. J.P.S., N.A., J.B., and P.S.S.: contributed to the acquisition of data, drafted the article, and final approval of the version to be published. M.C.: contributed to the conception and design of the work, drafted the article, revised it critically, and provided final approval of the version to be published. M.B.: contributed to the conception and design of the work, acquisition of data, analysis and interpretation of data, drafting the article, and revising it critically, and final approval of the version to be published.

## Conflicts of Interest

The authors declare no conflicts of interest.

## Data Availability

The data that support the findings of this study are available from the corresponding author upon reasonable request.
